# Affordable lab-scale electrospinning setup with interchangeable collectors for targeted fiber formation

**DOI:** 10.1016/j.ohx.2023.e00501

**Published:** 2023-12-12

**Authors:** Alexi Switz, Aditi Mishra, Katrina Jabech, Anamika Prasad

**Affiliations:** a Department of Biomedical Engineering, Florida International University, Miami, FL, United States; b Department of Mechanical and Materials Engineering, Florida International University, Miami, FL, United States

**Keywords:** Electrospinning, Rotating drum, Coiled fiber, Directed fiber, Tissue engineering

## Abstract

The electrospinning method is increasingly in demand due to its capability to produce fibers in the nanometer to micrometer range, with applications in diverse fields including biomedical, filtration, energy storage, and sensing. Many of these applications demand control over fiber layout and diameter. However, a standard flat plate collector yields random fibers with limited control over diameter and density. Other viable solutions offering a higher level of control are either scarce or substantially expensive, impeding the accessibility of this vital technique. This study addresses the challenge by designing an affordable laboratory-scale electrospinning setup with interchangeable collectors, enabling the creation of targeted fibers from random, aligned, and coiled. The collectors include the standard flat plate and two additional designs, which are a rotating drum and a spinneret tip collector. The rotating drum collector has adjustable speed control to collect aligned fibers and exhibits stability even at high rotational speeds. The spinneret tip collector was designed to produce helically coiled fibers. The setup was validated by directed fiber formation using polycaprolactone (PCL), a biodegradable and FDA-approved polymer. Overall, the uniqueness of the design lies in its affordability, modifiability, and replicability using readily available materials, thus extending the reach of the electrospinning technique.

## Graphical abstract

**Figure:**
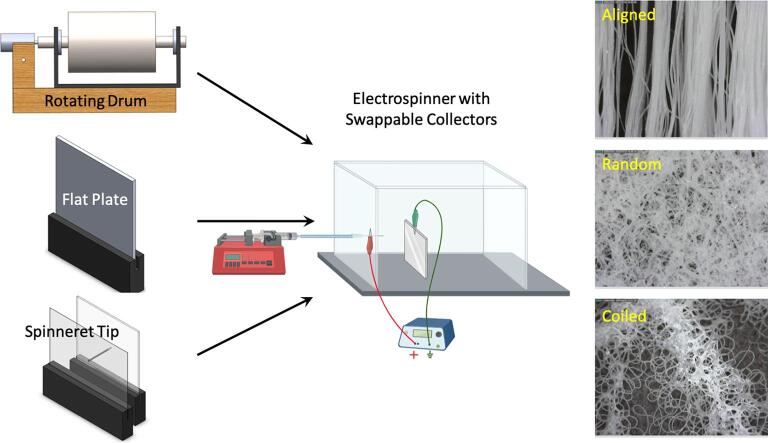

**Specifications table**

**Table:** 

Hardware name	Electrospinning Setup with Various Collectors
Subject area	Materials Science, Biomedical Materials
Hardware type	Manufacturing
Closest commercial analog	Spingenix SG100-CCS1000
Open source license	This work is licensed under a Creative Commons Attribution 4.0 International License.
Cost of hardware	$4,100
Source file repository	https://doi.org/10.17632/cyb36wsh6c.2

## Hardware in context

Nanofibers have emerged as materials of interest in the past few decades for multiple applications, including tissue engineering, food packaging, energy, and sensor applications due to their versatile properties, such as the ratio of high surface area to volume, porosity, and versatility of material choices available [1], [2], [3], [4], [5]. Electrospinning is the most widely used synthesis technique for nanofiber formation due to its versatility in fiber formation of various diameters and designs, scalability, and compatibility for an extensive range of materials. However, while electrospinning requires a simple setup, limited commercial options are available and are costly. Furthermore, since electrospinning is an area of active research, incorporating new emerging techniques requires modifications that are either expensive or not possible due to the closed design of commercial setups. Hence an open-source affordable design can significantly advance technology and accessibility, as has been the case when open sources became available for 3D printing. The above limitation is addressed by this study by designing an affordable electrospinning setup to spin controlled fibers and is accessible for future modifications.

Controlled fiber morphology and design for electrospun mats find several uses. Aligned nanofibers are of interest for their enhanced structural strength along the deposition direction and for their functional needs, such as guided cell migration and growth and improved energy storage efficiency [6], [7], [8]. Helically coiled nanofibers are commonly present in natural systems [9], [10] but are less reported in engineered systems. Helical coiling allows for higher porosity, larger surface area, mechanical flexibility, and toughness than their linear counterparts, making them suitable for filters, sensors, energy storage, actuators, drug delivery, and tissue engineering applications [11], [12].

The nozzle design plays an important role in process stability and the collector plays the most significant role in the structural morphology of the electrospun mats. A 3D printable nozzle with automation in process parameters was recently reported [13]. In terms of collector design, electrospinning hardware typically accommodates one collector, either a stationary flat plate collector or a rotating drum collector [13]. Stationary flat plate collectors gather nanofibers on flat surfaces in an entangled, randomly oriented direction. A rotating drum collector design is one of the most upfront and efficient methods to obtain well-aligned nanofibers [14]. However, the collection of coiled fibers has been less developed. A spinneret tip collector has been reported to produce coiled fibers due to a concentrated electric field around the tip [15]. The proposed electrospinning setup allows for the creation of all the above types of fiber morphology by incorporating three different types of collector design while remaining cost-effective.

While electrospinning is not a new technology, there are limited suppliers and even limited availability of cost-effective laboratory setups, thus restricting the widespread use of electrospinning technology. Basic electrospinning research setups with flat plate collector design typically range from $10,000 to $50,000, depending on enclosure needs. The cost further rises to $100,000 and more with modifications in collector and tip designs. To provide alternative solutions for cost-effective electrospinning technology, in our previous work, we developed an in-house electrospinning platform with a static collector for research [16] and a mobile platform with a rotating drum collector for classroom demonstration [17]. The current paper significantly improves the research setup with multiple collector design, safety enclosure, and replication details, thus improving both the design and its accessibility.

Specifically, the current paper presents the design, manufacturing details, and comprehensive part list for three different collectors: the flat plate, rotating drum, and spinneret tip collector. The report also demonstrates the effectiveness of the setup via fiber formation and its characterization using PCL, a biodegradable polymer with widespread application [18], [19]. The paper provides a detailed blueprint for replicating the design for widespread use and developments in electrospinning research.

## Hardware description

**Fig. 1:**
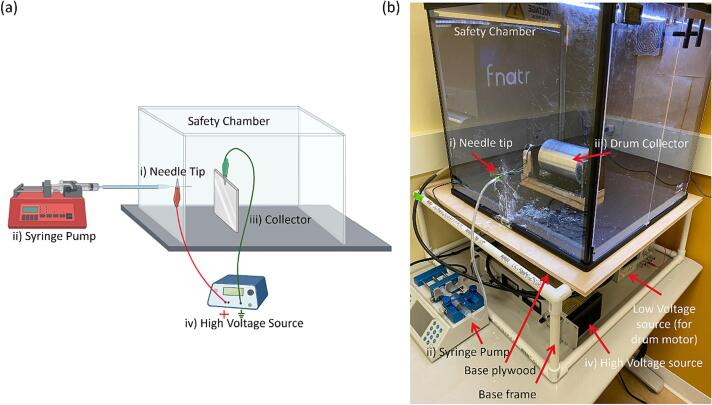
Electrospinning setup showing (a) general schematic (b) assembled setup with a drum collector plate. In both the figures, the key components are marked, which include: (i) metallic needle tip, (ii) syringe pump for driving controlled fluid, (iii) collector, and (iv) high voltage power source for voltage drop between the needle tip and collector. A low-voltage source is additionally needed for using the rotating drum collector.

The key components of a basic electrospinning setup are shown schematically in Fig. 1a and includes: (i) a syringe with a metallic needle tip, (ii) a syringe pump to drive fluid through the syringe at a controlled rate, (iii) a collector for fiber deposition, and (iv) a high-voltage power supply to apply voltage drop between the needle tip and collector. Additional components are needed based on the collector. For example, a low-voltage power source is required for running the motor of the drum collector. The components are typically enclosed within a cabinet for safety and environmental controls.

The base setup with a flat plate can produce fibers from nano to micrometer range via the control of process parameters (such as voltage, flow rate, and needle-to-collector distance), fluid properties (such as viscosity, surface tension, and molecular weight), and ambient parameters (temperature, humidity) [20], [21]. Additionally, two new collector designs, namely a rotating drum, and a spinneret tip collector, dictate fiber morphology from aligned to helically coiled structures. The forces in the rotating drum collector tend to wind the fiber to the surface of the rotating drum, thus producing aligned nanofiber mats [22]. Additional process controls include rotating speed, where increasing the speed tends to stretch the fiber, aiding in reducing fiber diameter. The spinneret tip collector results in an electrical field concentrating around the tip, resulting in a coiled fiber [15].

The assembled design of the current work is shown in Fig. 1b with the rotating drum collector. All the CAD files were designed using SolidWorks (Dassault Systèmes, USA) and where relevant printed using the Creality Ender 5- Plus printer.

**Fig. 2:**
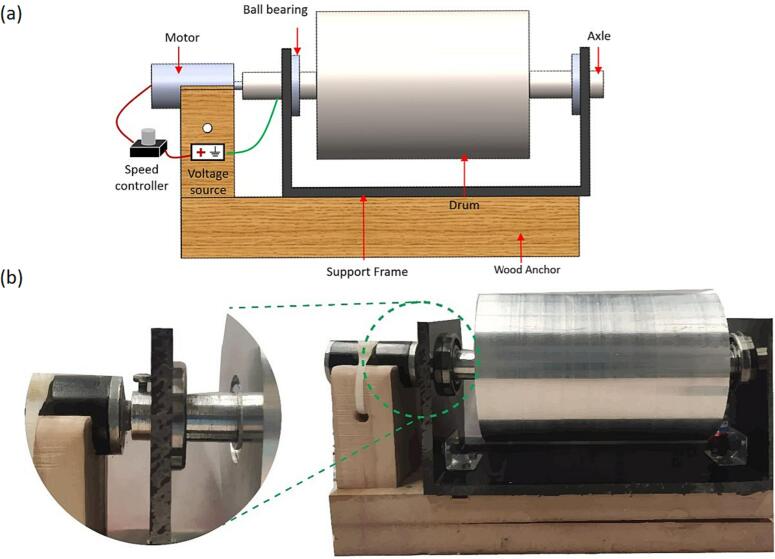
Rotating 3D drum collector with (a) CAD model showing the roller drum, a 2-inch axle, end ball bearings, a DC motor for driving the system, a support frame, and (b) as-fabricated drum collector. A low-voltage power source (not shown here) drives the motor. The drum and axle are made from aluminium and the frame is made from Polypropylene. The aluminium components (axle and drum) were fabricated using a lathe. The unit was anchored and tied down on a wooden base for structural support needed at the high rotation speed.

A rotating collector consists of a drum connected to a DC motor capable of rotating the drum at variable speeds (100–4000 rpm). Fig. 2a shows the CAD model of the design, and Fig. 2b shows the assembled drum. The design uses a conductive metal drum mounted on an insulating support. The conductive axle is threaded inside the drum for rotation and is connected to a 24 V high-speed DC motor via a ball bearing for driving the system. The motor is connected to a speed controller, operated using a 24-V power source.

The design feature separating the drum from the axle is an additional feature for design adaptability for accommodating drums with different outer diameters on the same frame. The above will reduce the cost and time of manufacturing the entire assembly for a different drum diameter. In terms of material, we used aluminium for the drum and axle due to its cost, and ease of machining. The limitation of using a solid aluminium drum was the high weight which required additional stabilization with the wooden base at high speeds. An alternative design can replace it with a lower diameter drum or polymer or wood wrapped with a conductive metallic foil to reduce the weight.

**Fig. 3:**
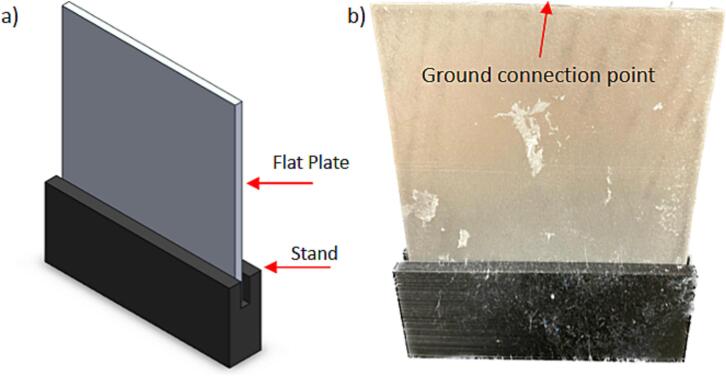
Flat collector with (a) CAD model showing the isometric view of the assembly with a flat plate mounted in a stand and (b) as-fabricated collector.

The flat plate collector assembly requires a grounded conductive plate and a non-conductive support to hold it vertically. Fig. 3a shows the CAD model of the design and Fig. 3b shows the as-fabricated system. We used aluminum as a conductive plate and 3D-printed polylactic acid (PLA) for the supporting stand, though alternative materials can be used.

**Fig. 4:**
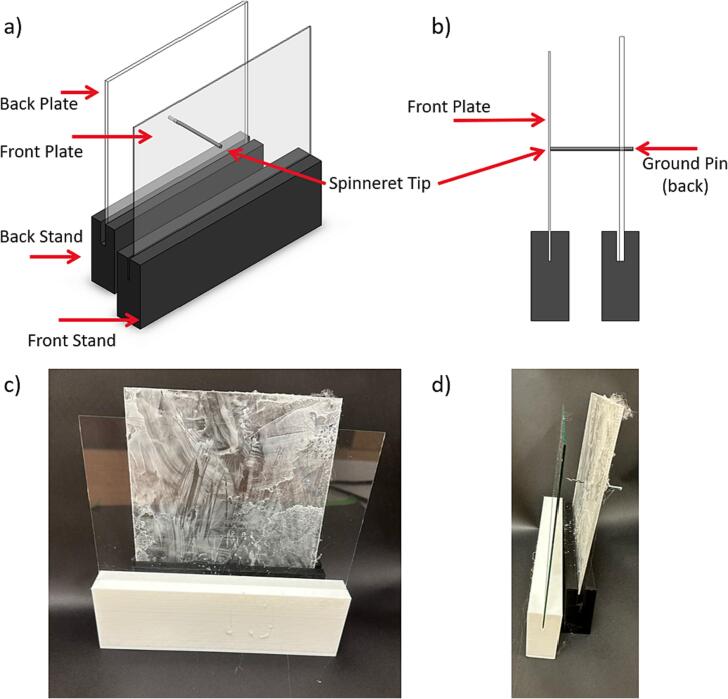
(a and b) CAD model showing spinneret tip collector mounted in stands, and (c and d) as-fabricated spinneret tip collector assembly.

The spinneret tip collector comprises a metal needle tip that is grounded, and two non-conductive flat plates that are placed directly in front of this needle tip. Fig. 4a shows a CAD model of the spinneret tip collector design and the fabricated system in Fig. 4b. We used glass as the front collector plate and the acrylic plate as the back plate to hold the needle tip. A hole was drilled in the acrylic plate at the desired height to secure the metal tip in place. The needle tip passed through this hole in the acrylic plate, ensuring only a small portion of the metal spinneret tip is exposed from the back of the plate. The tip rests at the back of the glass plate but does not pierce the glass plate. We 3D-printed polylactic acid (PLA) for the supporting stands, though alternative materials can be used.

### Final assembly

Fig. 1b shows the final assembly of the design. The syringe pump used is Fusion 101A (ChemyX, USA) which has a maximum flow rate of 128 mL/sec and a step resolution of 0.046 μm. The high voltage source is H030HP1 (Acopian, USA), with a maximum output of 30KV. A cost-effective plexiglass enclosure designed for 3D printers was purchased on Amazon (FNATR Box) and comes with LED light, HEPA Filter Fan, and thermal and hydration sensor. The enclosure is also needed for proper wire management, protection from outside forces, and a directed space for fiber deposition. In its absence, the fibers can get collected on other exposed surfaces, such as the surface of high-voltage wires. The enclosure is placed on top of a base assembly made from PVC pipe and plywood, as shown in the figure.

The syringe is placed on the pump to push desired fluid through at controlled rates and is connected to the needle tip via tubing with a 4 mm inner diameter. The needle tip is held in place by a hole drilled into the enclosure wall. The tip is connected to the positive end of the high-voltage power source using an alligator clip. Multiple collectors can be used with the system depending on fiber requirement. The chosen collector is placed inside the enclosure at a suitable height and distance from the needle tip, and the ground is connected with the conductive end of the collector.

Fig. 1b shows the rotating drum collector inside the chamber, which can be replaced with other collector designs. When using the rotating drum collector, the drum is connected to its speed controller and 24-volt power supply for rotating the drum at a controlled speed. To use the flat plate collector, other collector assembly is removed and replaced by the flat plate assembly. A grounded alligator clip is attached at the center at the metal plate top, as indicated in Fig. 3b. The grounded static flat plate collector is placed inside the environmental chamber in the same setup as the rotating drum collector. Similarly, to use the spinneret tip collector, any existing collector assembly is removed and replaced by the spinneret tip collector setup. The grounded alligator clip is attached to the back of the metal spinneret tip, as indicated in Fig. 4b.

### Key aspects of the use of the hardware

The electrospinning setup is valuable for researchers that require electrospun fibers with specified properties across different applications. Examples are scaffolds for biomedical applications and conductive fibrous mats for sensor applications. The designed hardware has multiple advantages over commercially available electrospinner setups and other in-house built systems listed below, making it a valuable and extensible tool for researchers from many fields.

• The hardware comes with three types of swappable collector plate designs, providing a unique design for producing a variety of fiber sizes and morphology tailored to specific needs.
• The rotating drum collector is designed to have swappable drums, another unique feature allowing researchers to accommodate different diameter drums for varying design needs.
• This hardware is compatible with a nearly endless range of polymer solutions.
• Together with material capability and changes in electrospinning parameters of the hardware (voltage, collector distance, rotating drum speed, controlled environment), the hardware can create fibers with diameters from the scale of nanometers to micrometers.
• In contrast with other in-house electrospinning designs, the assembly can easily be replicated since it uses readily available components sourced from large-scale retailers.
• This hardware is relatively compact and can be set up on any sturdy table with access to outlets.
• This hardware is at least one order of magnitude lower in cost than commercial options and allows endless design modifications for the future.

### Build instructions

The hardware description and related schematic provide details of the hardware assembly, the material used, and additional instructions in choosing alternative materials depending on the availability of resources. Below is build instruction for the design shown in Fig. 1 using selected materials from Table 2. The tools required for the assembly include hand tools (screwdriver, pipe cutter), access to a machine shop (lathe and bandsaw), and access to a 3D printer (such as Creality Ender). The instructions are below.

1. Download all CAD files (links in Table 1).
2. Rotating drum collector base.
  a. Cut the polypropylene sheet to form an 8 in. x 3.75-inch plate to form the base of the rotating drum collector (Fig. 5a). This base needs to be nonconductive, therefore polypropylene was chosen.

**Fig. 5:**
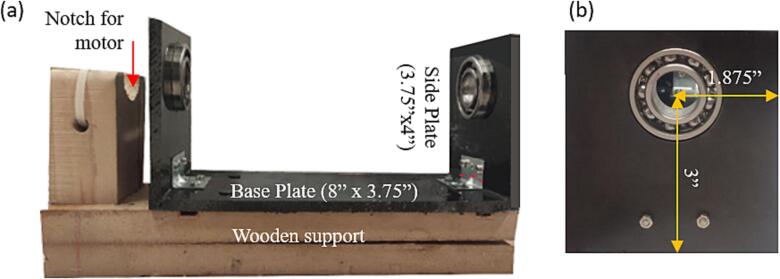
Components of the frame, which include (a) base Polypropylene frame mounted on a wooden support, (b) the side plate showing the pressure-fitted ball bearing.
  b. Cut the polypropylene sheet to form two 4 in. x 3.75-inch plates for holding the ball bearings and the axles (Fig. 5b).
  c. Use a drill to cut a 42 mm hole in each of the side plates 1.875 in. from the side and 3 in. from the bottom of each plate, for the ball bearings as shown in the rotating drum collector CAD file (Fig. 5b).
  d. Use glue and the corner brackets to connect the base and side plates (Fig. 5a).
  e. Attach the ball bearings to the polypropylene side plate support by pushing them through the predrilled holes with a press (Fig. 5b).
  f. *Optional:* If using a wooden support to stabilize the drum assembly, cut the wooden support to size as show in the rotating drum cad file. The wooden support will consist of two pieces glued together. The first piece is a 10.75-inch x 3.475-inch x 1.625-inch rectangle. The second piece is a 3-inch x 2.4375-inch x 1.4375-inch rectangle. The smaller rectangle piece will be glued to the larger rectangle as shown in Fig. 5a. The wooden support is used to add additional stability to the rotating drum at high rotating speeds, if operating the drum at lower speeds or if using a lower weight drum, this additional support may not be necessary.
  g. *Optional:* If using a wooden support stabilize the drum assembly, cut a semicircular notch with a diameter of 26 mm in the smaller rectangle wooden support to hold the motor. Drill a 0.125-inch hole beneath the notch to allow for a zip tie to be threaded through the hole and over the top of the motor to hold the motor in place (Fig. 5a).
  h. *Optional:* If using the wooden support, glue the polypropylene base to the wooden support pieces (Fig. 5a).
3. Rotating drum collector cylinder
  a. Use a lathe to smooth the 4-inch aluminium cylinder surface (Fig. 6a). This rotating drum collector allows for the use of interchangeable drums on the same base structure, the drum could be made of a variety of conductive materials. We utilized aluminium because it is affordable and easy to machine, one limitation of the aluminium drum is the high weight. To overcome this, a plastic or wooden drum could be machined and then a conductive foil could be used to cover the drum.

**Fig. 6:**
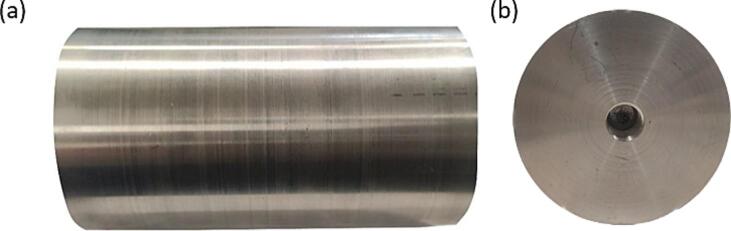
Components of the drum, which include (a) the front view of the Aluminium drum after smoothing to the required diameter, and (b) side view of the drum showing the internal threading at the center.
  b. Cut a 0.75-inch diameter hole 0.75-inch deep into the center of each side of the 4-inch aluminium cylinder (Fig. 6b).
  c. Put female threading on both ends of the 4-inch diameter rotating drum in the holes made in the previous step (Fig. 6b). Ensure the threading on both sides is towards the same direction to prevent the axles from coming out during rotation.
  d. Use the 0.75-inch aluminium cylinder and cut it into two equal pieces 2-inch-long pieces to form the axles. (Fig. 6c). The outer diameter of the axle needs to be the same as the inner diameter of the ball bearing. If the diameters do not match up, machine the axle to fit properly.
  e. Put 0.75-inch of male threading on one end of each of the 0.75-inch aluminium cylinders (Fig. 6c).
  f. On both axles, on the side opposite of the male threading, make a hole in the axle face to match the motor’s nozzle, as shown in Fig. 7.

**Fig. 7:**
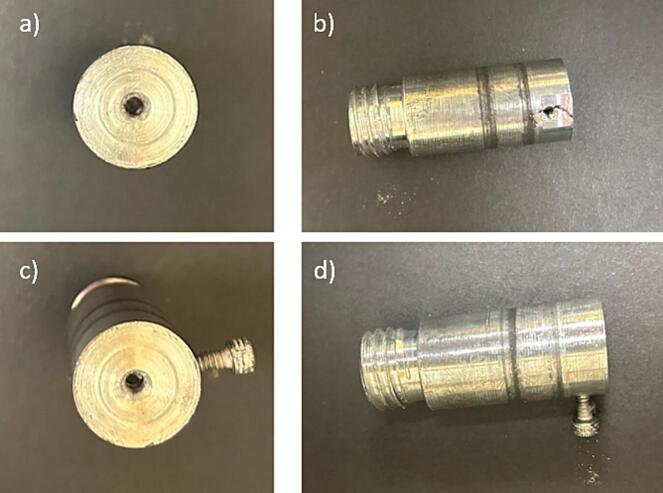
Components of the drum-axle, which include (a) the top view of the Aluminium axle with hole for motor attachment, used on both ends of the drum, (b) side view of the axle showing the threading’s and hole for bolt, (c) the top view of the Aluminium axle with bolt in place, used on both ends of the drum, and (d) the side view of the axle with bolt in place.
  g. On both axles, on the side opposite of the male threading, make an additional hole, 1/8th-inch from the end of the axle on the side with threading to fit a bolt, as shown in Fig. 7.
  h. Attach the motor to the aluminium drum axle using the hole made to match the motor’s nozzle. Ensure the motor nozzle is sufficiently inside the axle prior to spinning to prevent slippage and damage to the motor (Fig. 8).

**Fig. 8:**
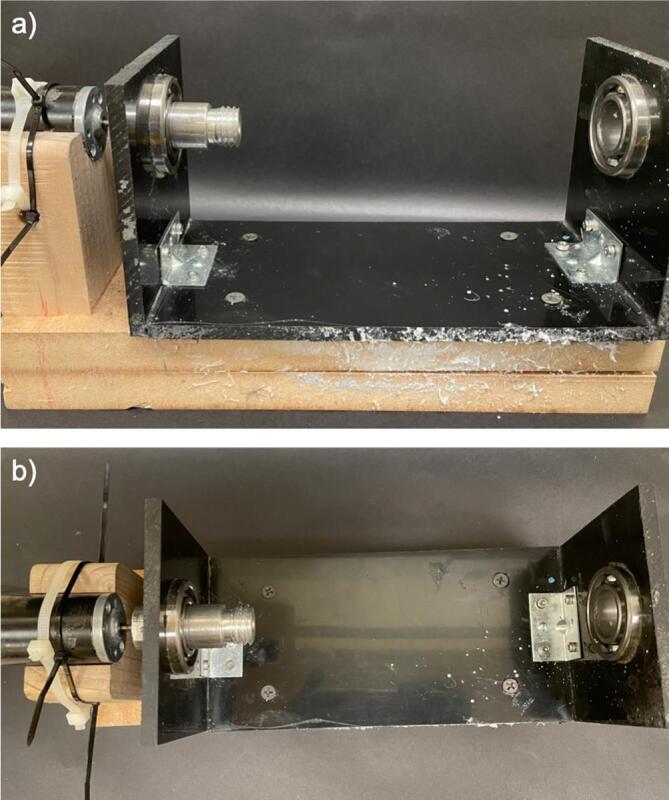
Rotating drum stand with motor and axle in place a) front view, and b) top view.
  i. Screw the axles to the drum on one end and secure in the ball bearings on the other end, securing the assembly in the support frame (Fig. 9a).

**Fig. 9:**
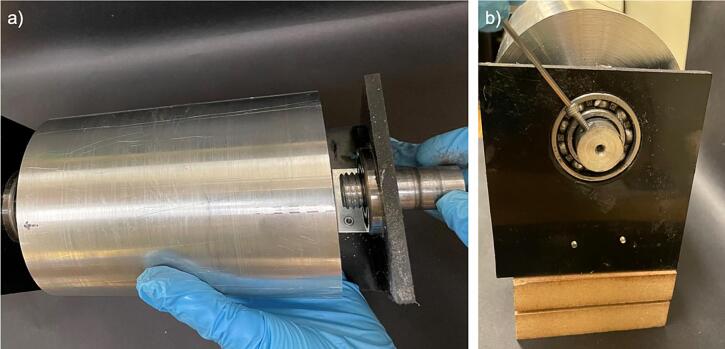
Rotating drum stand with drum and axle in place a) connecting the drum to the axle, and b) securing the axle in place with bolt.
  j. Attach a bolt to each axle on the outer side of the ball bearing and polypropylene base to secure the axle in place (Fig. 7, Fig. 9b).
  k. *Optional:* You may use an additional mechanism to stabilize the drum and motor. Here we used wood support and zip tie as one possible mechanism, as shown in Fig. 8a.
4. Flat plate collector
  a. Use the PLA filament and related CAD file to 3D print the flat plate stand on a 3D printer of your choice (Fig. 10). The stand is 15.5 cm long to span the length of the flat plate, 2.8 cm wide and 6 cm tall to provide stability to hold the plate. A 2 cm slit comparable to the plate thickness was provided to hold the plate tightly at a 90° angle.

**Fig. 10:**
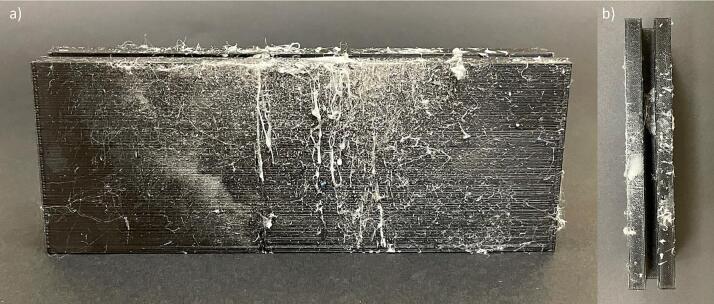
Flat plate collector stand 3D printed using PLA filament showing the (a) front and (b) top view (the white residue on the stand is from spinning and should be ignored).
  b. Cut the 6 mm aluminium flat metal sheet to the appropriate size (15 cm x 15 cm) for the flat plate collector (Fig. 11). These dimensions were chosen based on the available materials and height of the needle tip. Different dimensions can be used based on available materials. If using different dimensions, change the dimensions of the flat plate stand to accommodate this change.

**Fig. 11:**
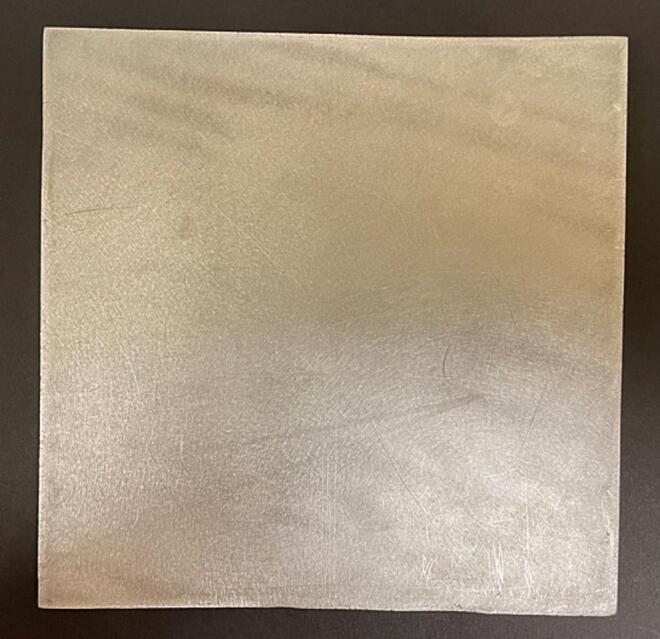
Flat plate collector made from metal plate of dimension 15 cm x 15 cm cut from a 6 mm thick aluminium sheet.
  c. Place the cut plate inside the groove of the stand to assemble the plate with the stand.
5. Spinneret tip collector
  d. Use the PLA filament and related CAD files to 3D print the two spinneret tip collector stands on a 3D printer of your choice (Fig. 12). Each stand has a slit comparable to the plat thickness to hold the plates tightly at a 90° angle.

**Fig. 12:**
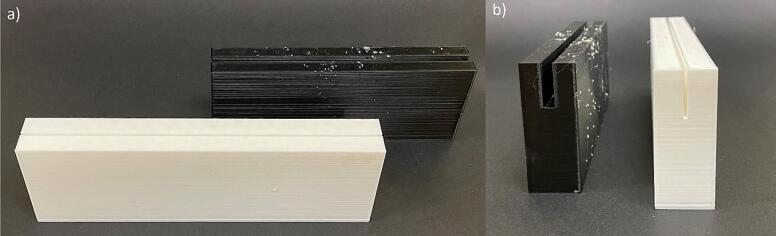
Spinneret tip collector stands showing the (a) front and (b)side view.
  e. Use an acrylic sheet and cut to an appropriate size (15 cm x 15 cm x 3 mm) for the spinneret tip back plate (Fig. 13a)

**Fig. 13:**
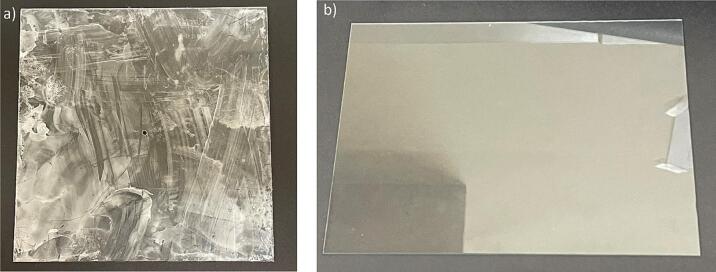
Spinneret tip collector plates, (a) acrylic back plate, with a central hole drilled to accommodate the spinneret tip, and (b) glass front plate.
  f. Drill a hole in the acrylic plate at the desired height of the spinneret tip and put the metal spinneret tip into the hole, so half of the metal spinneret tip is in front of the plate and half of the metal spinneret tip is behind the plate (Fig. 13a and 14).
  g. Place the acrylic plate on the black stand. It will be used as the back plate (Fig. 14).

**Fig. 14:**
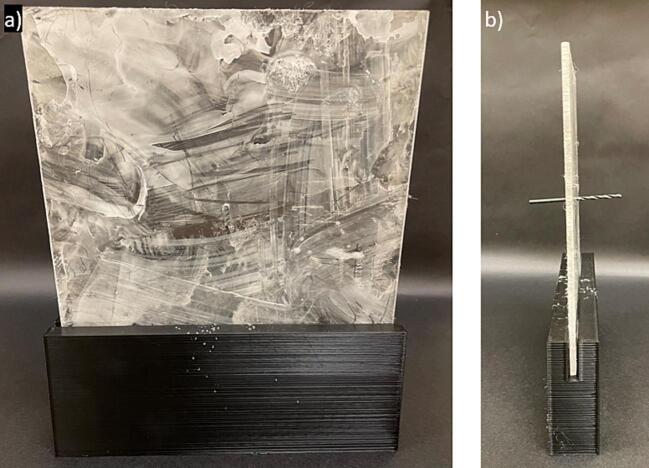
Spinneret tip collector acrylic plate with spinneret tip, (a) front view, and (b) side view.
  h. The size of the glass plate can be changed to collect different sized mats, we used a precut 5-inch x 7-inch glass plate. Any glass picture frame cover can be used for this front plate. If using a different size glass plate, change the dimensions of the glass plate stand to accommodate this change (Fig. 13b).
  i. Use the glass plate (Fig. 13b) as the front plate, place it in its stand at an appropriate distance, and assemble it as per Fig. 4a and Fig. 15. Ensure the metal spinneret tip is touching the front glass plate (Fig. 15b).

**Fig. 15:**
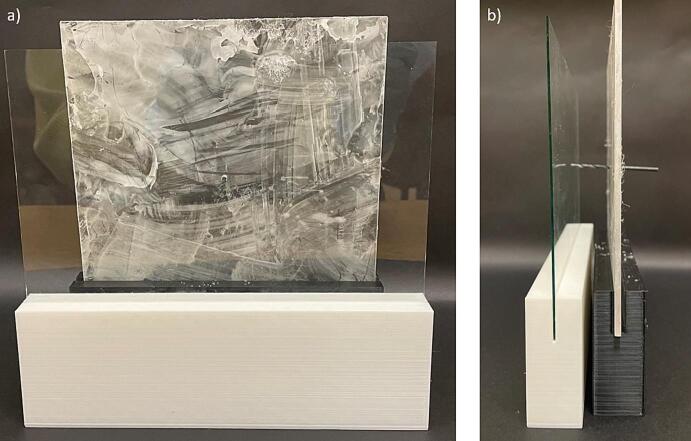
Spinneret tip collector, (a) front view, and (b) side view.
6. Assembly
  a. Use PVC pipes and PVC pipe connectors to form a square frame of 26-inches x 26 in. roughly 10-inches tall. These dimensions can be modified to accommodate space limitations, but the base should be large enough to hold the safety chamber (Fig. 16).

**Fig. 16:**
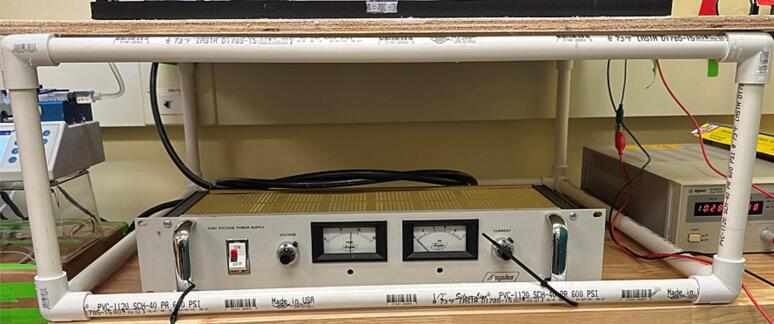
PVC frame with plywood board to serve as stand, with high voltage power source below and low voltage power source to the right.
  b. Cut the plywood and place on top of the pipe frame (Fig. 16).
  c. Assemble the safety chamber as per instructions with purchase and put the assembly on the pipe frame base (Fig. 17).

**Fig. 17:**
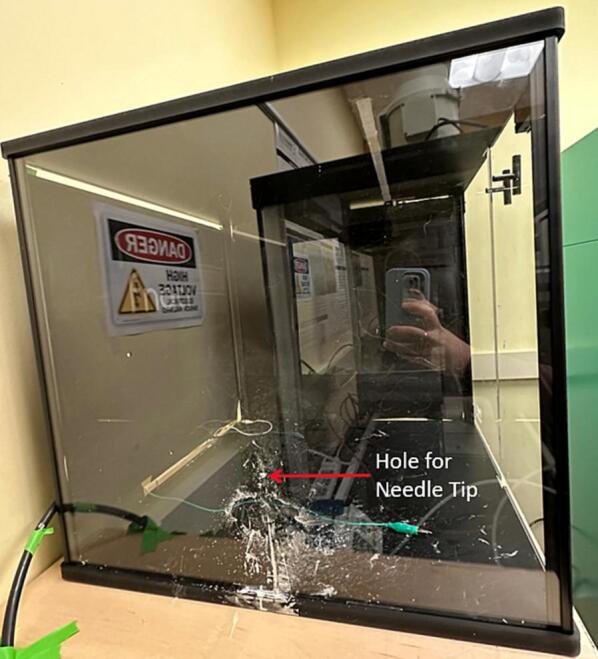
Safety chamber with hole drilled to accommodate needle tip.
  d. After the safety chamber is assembled, use a drill to make a small hole in the wall of the safety chamber closet to the side for the needle tip. The hole can vary in size depending on the needle tip used (Fig. 17)
  e. *Optional:* We have chosen to cover the clear acrylic safety chamber with black paper to better visualize the Taylor cone and fibers formed. We did this by cutting black paper to size and then taping it onto the outside of the safety chamber (Fig. 18)

**Fig. 18:**
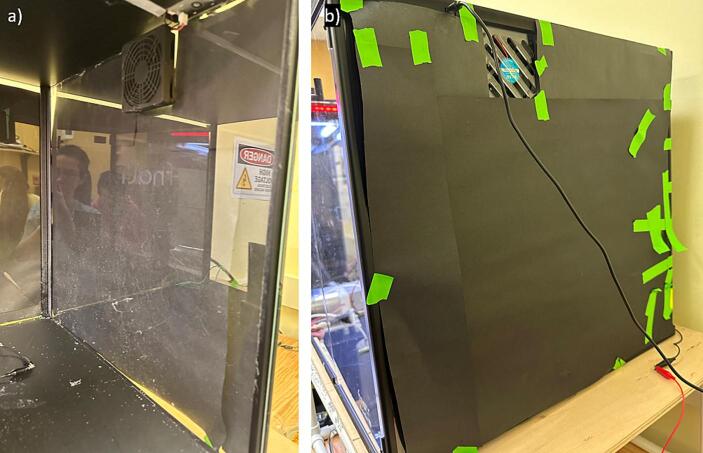
Environmental chamber covered in black construction paper a) interior view, and b) exterior view.
  f. Place the pump on one side of the safety chamber.
  g. Place the high-voltage and low-voltage power sources below the space created by the pipe frame, as shown in Fig. 16.
7. Electrical connection
  a. Connect the positive and negative low-voltage power outputs to the motor controller and the ground output to the collector (Fig. 19).

**Fig. 19:**
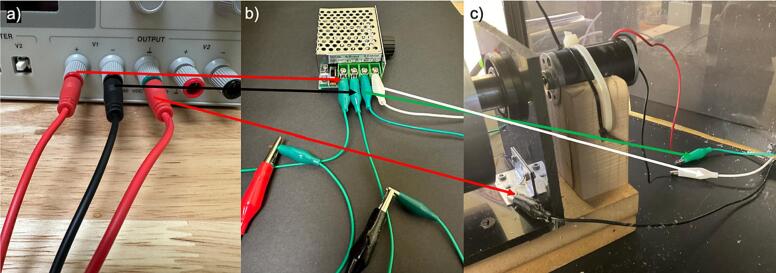
Low voltage power supply electrical connections a) power cables connections to low voltage power supply, b) low voltage power supply + and – outputs connected to motor controller, and c) motor controllers + and – outputs connected to motor and ground connection to rotating drum.
  b. Connect the high-voltage power source using instructions that come with that equipment. For electrospinning, the high voltage end with the needle tip using an alligator clip (Fig. 20).

**Fig. 20:**
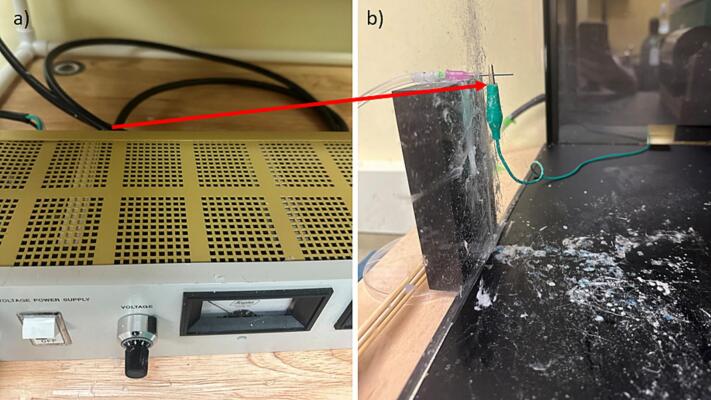
High voltage power supply electrical connection a) power cable connection to high voltage power supply, and b) high voltage power supply connected to needle tip.

**Table 1:** The table detailing the files used to assemble the electrospinning platform with hyperlinks to download the files.

**Design files summary**			
Design file name	File type	Open source license	Location of the file
Rotating drum- Assembly (Fig. 2a)	CAD	CC BY 4.0	https://doi.org/10.17632/cyb36wsh6c.2
Flat plate- Assembly (Fig. 3a)	CAD	CC BY 4.0	https://doi.org/10.17632/cyb36wsh6c.2
Spinneret- Assembly (Fig. 4a)	CAD	CC BY 4.0	https://doi.org/10.17632/cyb36wsh6c.2
Rotating Drum- Drum(Fig. 2a)	CAD and STL	CC BY 4.0	https://doi.org/10.17632/cyb36wsh6c.2
Rotating Drum- Motor(Fig. 2a)	CAD and STL	CC BY 4.0	https://doi.org/10.17632/cyb36wsh6c.2
Rotating Drum- Wood Anchor(Fig. 2a)	CAD and STL	CC BY 4.0	https://doi.org/10.17632/cyb36wsh6c.2
Rotating Drum- Ball Bearings(Fig. 2a)	CAD and STL	CC BY 4.0	https://doi.org/10.17632/cyb36wsh6c.2
Rotating Drum- Axles (Fig. 2a)	CAD and STL	CC BY 4.0	https://doi.org/10.17632/cyb36wsh6c.2
Rotating Drum- Support Frame (Fig. 2a)	CAD and STL	CC BY 4.0	https://doi.org/10.17632/cyb36wsh6c.2
Flat Plate- Metal Plate (Fig. 3a)	CAD and STL	CC BY 4.0	https://doi.org/10.17632/cyb36wsh6c.2
Flat plate- Stand (Fig. 3a)	CAD and STL	CC BY 4.0	https://doi.org/10.17632/cyb36wsh6c.2
Spinneret- Glass Plate (Fig. 4a)	CAD and STL	CC BY 4.0	https://doi.org/10.17632/cyb36wsh6c.2
Spinneret- Glass Plate Stand (Fig. 4a)	CAD and STL	CC BY 4.0	https://doi.org/10.17632/cyb36wsh6c.2
Spinneret- Acrylic Plate (Fig. 4a)	CAD and STL	CC BY 4.0	https://doi.org/10.17632/cyb36wsh6c.2
Spinneret- Acrylic Plate Stand (Fig. 4a)	CAD and STL	CC BY 4.0	https://doi.org/10.17632/cyb36wsh6c.2
Spinneret- Spinneret Tip (Fig. 4a)	CAD and STL	CC BY 4.0	https://doi.org/10.17632/cyb36wsh6c.2

**Table 2:** Materials used to assemble the electrospinning platform and their cost (Hyperlinks are included in the Specifications table). The total cost of these comes to 4093.61 USD.

Bill of materials summary						
Designator	Component	Number	Cost per unit [USD]	Total cost [USD]	Source of materials	Material type
Rotating drum, drum (4-inch dia x 5.75 in. long)	6061-T6511	1	36.00	36.00	Midwest Steel and Aluminium link	Aluminum
Rotating drum, axle (0.75-inch diameter x 4-inch long)	6061-T6511	1	8.48	8.48	Midwest Steel and Aluminium link	Aluminum
Rotating drum, motor (24 Volt High Speed 4000 RPM)	B078GJSK4R	1	28.88	28.88	Amazon link	Other
Rotating drum, power supply for motor	E3620A*	1	1099.00	1099.00	Digikey Link	Other
Rotating drum, speed controller	B071NQ5G71	1	15.99	15.99	Amazon link	Other
Rotating drum, ball bearing (20 mm Bore, 42 mm OD)	WBB973124	2	4.96	9.92	Global industries link	Metal
Rotating drum, support frame polypropylene sheet	NA	1	32.38	32.38	Interstate Plastic link	Polypropylene
Rotating drum, support frame adhesive	B07SWTPWRM	1	5.49	5.49	Amazon link	Glue/ Epoxy
Rotating drum, support frame corner brackets (1.5 in x 0.75 in x 0.75)	809432	1	3.48	3.48	Lowes link	Steel
Flat plate, metal plate (6 mm thick)	CL002-A3-3 T	1	28.00	28.00	Bulkman link	Aluminum
Spinneret tip, front glass plate	B0777JF4YZ	1	5.99	5.99	Amazon link	Glass
Spinneret tip, back acrylic plate sheet	BOB5KQBVL1	1	22.99	22.99	Amazon link	Acrylic
Spinneret tip, collector nails	B08MQP6252	1	12.99	12.99	Amazon link	Stainless Steel
3D filament for collector stand		1	26.99	26.99	Amazon link	Polylactic acid
Assembly, high voltage source	P030HP1*	1	1460.0	1460.0	Acopian link	Other
Assembly, syringe pump	10071*	1	999.0	999.0	Chemyx link	Other
Assembly, safety enclosure	B09QGCR548	1	199.0	199.0	Amazon link	Other
Assembly, electrical connections alligator clips	B0995KJWR5	1	5.99	5.99	Amazon link	Other
Assembly base, plywood	PLY-07-00179	1	29.98	29.98	Lowes link	Plywood
Assembly, PVC pipe 0.5 in.	PVC040050600	1	3.96	3.96	Lowes link	Polymer
Assembly base, PVC pipe 0.5-inch elbow connector	PVC025100600	8	2.58	20.64	Lowes link	Polymer
Consumables, syringes (10 mL)	B07KQP69RG	1	16.99	16.99	Amazon link	Polymer
Consumables, needles (1.5 Inch 14G)	B07H81211S	1	9.89	9.89	Amazon link	Metal
Consumables, pump tubing	B0002563MM	1	6.99	6.99	Amazon link	Polymer
Consumables, tubing connector	B08LN7MQ7X	1	4.59	4.59	Amazon link	Polymer

* Other cost-effective options for power supply and pump can be used [23] to bring them down further. The pump can also be constructed in-house [24] to reduce the cost.

*Caution: It is important to keep the high-voltage alligator clip separated from the motor controller alligator clips. It is beneficial to use holes in the safety chamber for wire management or drill additional holes as needed. We used the existing hole at the back to bring in the high-voltage alligator clips within the chamber to connect with the needle tip and collector.*

## Operation instructions

Table 2 also lists the hardware consumables for the operation of the machine, which includes a syringe, needle tip, and connector pipes. To operate the electrospinning setup, we first need to make a polymer solution for spinning. We utilized PCL dissolved in Dichloromethane (DCM or methylene chloride), the solution preparation instructions in Section “Solution Preparation and Fiber Formation”. However, a variety of other polymers and solvents can also be used. Instructions for operating the equipment are given below.

1. Prepare the polymer solution as per solution preparation instructions in Section “Solution Preparation and Fiber Formation”.
2. Cut the tubing to the proper length span between the syringe pump and the hole in the environment chamber cut out for the needle tip (approximately 12 in., but could be modified based on height of tables, stands, etc.)
3. Attach tubing to the syringe, on the other end of the tubing, attach the adapter and needle tip.
4. Fill the syringe with the polymer solution. It is easiest to do this when the tubing is attached as it reduces the amount of solution that will be lost during the transfer process (Fig. 21).

**Fig. 21:**
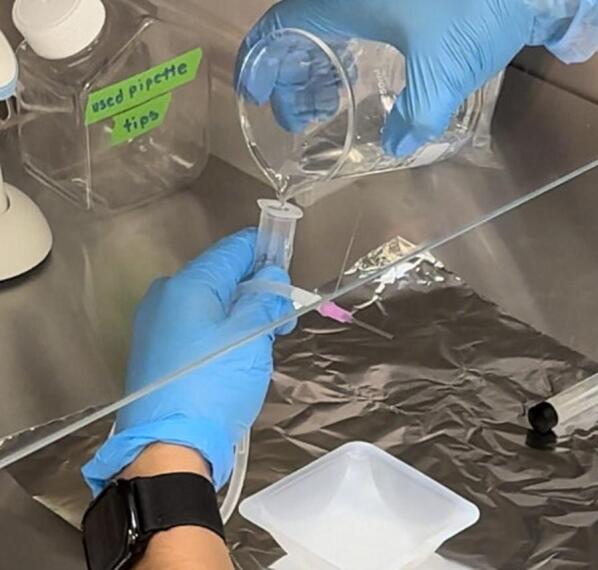
Syringe filling process.
5. Load the syringe in the pump and secure it in place using the syringe pump clamps (Fig. 22). Since each machine will have slightly different clamping instructions, use the instructions that come with the machine.

**Fig. 22:**
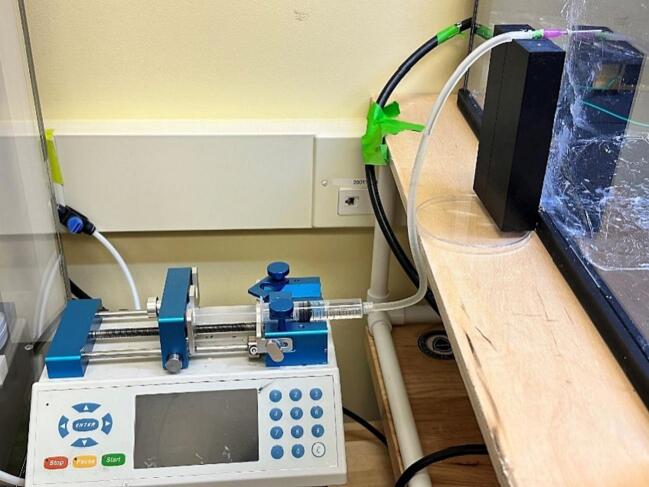
Syringe and needle tip placed in pump and environmental chamber.
6. Place the needle tip into the hole drilled into the safety chamber, the needle tip needs to remain connected to the adapter, tubing and syringe during this process (Fig. 22)
7. Attach the high voltage power alligator clip to the needle tip.
8. Place saran wrap over the base of the safety chamber to collect any potential drips produced by the needle tip.
9. Select the collector you would like to use and place the collector at an appropriate distance from the needle tip. The distance between the needle tip and the collector can change based on the desired fiber formation.
10. Attach the grounded connector clip to the ground connection point on the collector. The ground connection point for each collector is shown in Fig. 23 below.

**Fig. 23:**
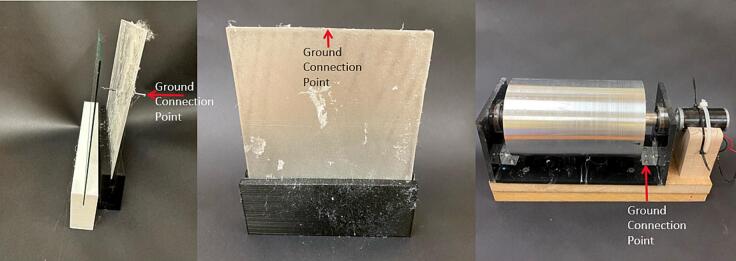
Ground connection points for each collector.
11. *Optional:* If the rotating drum is being used, connect the positive and negative alligator clips from the DC motor controller to the respective inputs on the rotating drum motor.
12. Once the electrospinning platform is set up, plug in the syringe pump and set the desired flow rate. The flow rate can be changed based on the desired fiber formation.
13. Put on voltage protective gloves before completing the next steps.
14. *Optional:* If the rotating drum is being used, plug in the low-voltage power supply, ensure it is set to 12 V, and use the DC motor controller to change the speed of the rotating drum as needed. Use a tachometer to confirm the rotation speed of the rotating drum (Fig. 24).

**Fig. 24:**
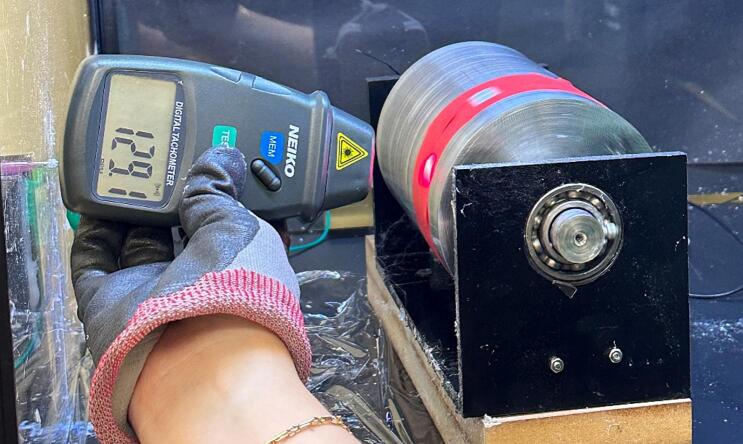
Using Tachometer to measure rotations per minute on rotating drum.
15. Press start on the pump. It will take time before the droplets start coming from the needle tip.
16. If not completed already via use of the rotating drum, plug in the low-voltage power supply, turn on the low voltage power supply to deliver the ground charge to the collector.
17. Plug in the high-voltage power supply and turn knob to select the appropriate voltage. The voltage can be changed based on the desired fiber formation.
18. Turn on the high-voltage power supply.
19. Step away from the safety enclosure.
20. Once the electrospinning process is complete (ie. Adequate fibers are formed, a change is necessary to the setup, electrospinning must be paused):
  a. Turn off the high voltage power supply.
  b. Turn off the low voltage power supply.
  c. Turn off the syringe pump.
  d. Remove alligator clips from connections.
  e. Remove collector and harvest fibers.
  f. Remove needle tip and syringe.

Important Safety Instructions

It is important to note that while the hardware is easy to assemble and use, as with any electrospinning setups, caution should be taken for safety as a high-voltage power supply is used. Some essential safety instructions are:

• Wire management: It is essential to keep the high-voltage line separate from the low-voltage cables and ground cables. Use zip ties as appropriate to manage and separate the wires.
• High voltage power sources: Always keep it unplugged when not in use and plug it only when ready to spin and all other connections are already made. Once the power supplies are turned on, do not touch any pieces of the electrospinning setup besides the on–off buttons on the power supplies.
• For safety concerns, wear a pair of rubber gloves before turning on the power supplies.
• Chamber cleaning: Clean the area post spinning and use a plastic drip cup below the needle to catch any liquid to keep the area clean.
• Please step away from the system when it is spinning but do not leave it unattended when in operation. You can keep an eye on using a digital camera.
• If the needle tip becomes clogged during the spin, turn off the power source and pump before approaching the assembly.

## Validation and characterization

To evaluate the performance of the setup, a PCL solution was created and electrospun on the three different collector designs. Since this electrospinning setup is proposed as a cost-effective method, we used a DinoLite Handheld microscope to image and analyze the fibers instead of conducting SEM imaging on the fibers. The DinoLite software can be downloaded online for free, and the microscope is USD 300 and can be ordered on Amazon.

### Solution preparation and fiber formation

The solution used for electrospinning was prepared using 17.5 wt% of PCL (Mn ∼ 80,000) purchased from Sigma Aldrich, dissolved in a solvent of DCM purchased from Carolina. Once dissolved, the solution was transferred into a 10 mL syringe with a 14-gauge blunt needle tip and electrospun at a voltage of 18 kV with a needle tip-to-collector distance of 15 cm. The same solution electrospinning variable was used to collect fibers for the three-collector setups with the same needle. The speed of the rotating drum was run at a constant rate of approximately 130 RPM as confirmed with a tachometer. The voltage for driving the motor was taken as 12 V.

**Fig. 25:**
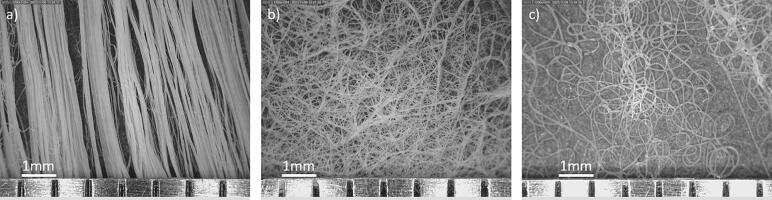
DinoLite images of fibers collected on (a) rotating drum, (b) flat plate, and (c) spinneret tip. All fibers were collected at 18 KV with the tip to collector distance of 15 cm. The fiber features were uniquely different on each collector, with aligned fibers from the drum, random from the flat plate, and coiled from the spinneret tip collector.

Fig. 25 depicts the typical fiber pattern obtained from each collector. The fibers are aligned in the direction of the rotation for the drum collector (Fig. 25a), random for the flat plate collector (Fig. 25b), and coiled on the spinneret tip collector (Fig. 25c). No beads were observed for the fibers collected on the rotating drum collector and flat plate collector. Some beads were observed for the fibers collected on the spinneret tip collector, but the overall structure of coiled geometry was obtained prominently, as observed in the figure.

Further modifications and additional studies will be conducted to continue improving material outcomes and upgrade to hardware design. The material outcome will focus on coiling diameter and coil sizing via altering parameters such as flow rate, voltage, and needle tip-to-collector distance. Further studies on the correlation of drum speed to the fiber diameter will also be conducted. Hardware upgrades will focus on adding the x-axis movement of the syringe or the flat plate collector to create mats of uniform thickness. While the temperature and humidity inside the safety chamber can be monitored, they cannot be directly controlled with this hardware, so designing a new chamber for the hardware is another aspect of the future.

## Summary and conclusion

Electrospinning is a crucial method for producing nanofibers for various applications. The paper introduces an affordable and versatile laboratory-scale electrospinning setup to address the limitations of existing commercial and in-house electrospinning setups. Specifically, it uses three interchangeable collector designs, namely a flat plate collector for random fibers, a rotating drum collector for aligned fibers, and a spinneret tip collector for helically coiled fibers. Each of the three collectors is validated using a biodegradable polymer PCL, to create nanofibers with controlled morphologies from random, aligned, and coiled.

Overall, the unique strength of the design lies in its affordability, ease of replication, and adaptability to modifications, making it a highly versatile tool for creating fiber morphologies across diverse applications. The open-source design provides a cost-effective platform for researchers to explore controlled nanofiber synthesis and paves the way for future advancements in electrospinning.

## License

This work is licensed under a Creative Commons Attribution-ShareAlike 4.0 International License.

## CRediT author statement

**Alexi Switz:** Spinneret collector conceptualization, Design and development, Device assembly, Material preparation, Writing. **Aditi Mishra:** Material Preparation, CAD Designs. **Katrina Jabech:** Material Preparation, CAD Designs. **Anamika Prasad:** Overall project and design conceptualization for the three collectors, Writing, Reviewing, and Editing.

## Declaration of competing interest

The authors declare that they have no known competing financial interests or personal relationships that could have appeared to influence the work reported in this paper.
